# Distinct phenotype of isolated dizziness stroke: association with cerebellar infarctions and elevated LDL-C

**DOI:** 10.3389/fneur.2026.1747076

**Published:** 2026-03-09

**Authors:** Bing Zhang, Xiaopeng Liu, Xiaohui Su, Lifang Chu, Hui Wang, Limin Nie, Shujing Yan

**Affiliations:** 1 Department of Neurology, Shijiazhuang People’s Hospital, Shijiazhuang, Hebei, China; 2 Department of Neurosurgery, The Second Hospital of Hebei Medical University, Shijiazhuang, Hebei, China; 3 Key Laboratory of Clinical Neurology, Ministry of Education, Hebei Medical University, Shijiazhuang, Hebei, China; 4 Department of Nephrology, Shijiazhuang People’s Hospital, Shijiazhuang, Hebei, China; 5 Department of Geriatrics, Shijiazhuang People’s Hospital, Shijiazhuang, Hebei, China

**Keywords:** acute ischemic stroke, cerebellum, infarction topography, isolated dizziness/vertigo, low-density lipoprotein cholesterol, posterior circulation stroke

## Abstract

**Background:**

Distinguishing ischemic stroke in patients presenting with isolated dizziness or vertigo (IDV) from more benign causes remains a significant clinical challenge. Current understanding of the specific clinical and imaging characteristics that differentiate IDV strokes from strokes with non-isolated symptoms (NIDV) is incomplete.

**Objective:**

This study aimed to systematically compare the clinical characteristics, with a specific focus on infarction topography and lipid profiles, between patients with acute cerebral infarction presenting with IDV and those with NIDV.

**Methods:**

In this retrospective cohort study, we analyzed 136 patients with Magnetic Resonance Imaging (MRI)-confirmed acute cerebral infarction who presented with dizziness/vertigo. Patients were classified into IDV (*n* = 53; NIHSS = 0, no focal deficits) and NIDV (*n* = 83; NIHSS>0 or focal deficits) groups based on a standardized neurological assessment. A comprehensive comparison of clinical characteristics was performed, including demographics, vascular risk factors, fasting lipid profiles, and neuroimaging features. Differences between groups were assessed using univariate analyses (Student’s *t*-tests, Chi-square tests, etc.), with variables significant at *p* < 0.10 eligible for inclusion in a multivariate logistic regression model to identify factors independently associated with the stroke phenotype (IDV vs. NIDV).

**Results:**

Univariate analysis revealed that the primary differences between groups lay in infarction topography and lipid profiles. Specifically, infarctions in the cerebellar hemisphere (47.2% vs. 25.3%; *p* = 0.009) and other cerebellar regions (18.9% vs. 4.8%; *p* = 0.009) were significantly more prevalent in the IDV group, whereas pontine infarctions were strongly associated with the NIDV group (13.2% vs. 41.0%; *p* = 0.001). Concurrently, the IDV group exhibited a more atherogenic lipid profile, with significantly higher levels of low-density lipoprotein cholesterol (LDL-C) (3.07 ± 0.89 vs. 2.71 ± 0.75 mmol/L, *p* = 0.013). Notably, the prevalence of acute lacunar infarcts was also higher in the IDV group (17.0% vs. 4.8%, *p* = 0.019). A history of hypertension was less prevalent in the IDV group (60.4% vs. 83.1%, *p* = 0.003), though this association was attenuated in the multivariate model (*p* = 0.052). In the multivariate model, pontine infarction remained a strong negative predictor of the IDV phenotype (adjusted OR = 0.30, *p* = 0.016), while a higher LDL-C level emerged as an independent positive predictor (adjusted OR = 1.67 per mmol/L, *p* = 0.036).

**Conclusion:**

In patients with confirmed acute cerebral infarction, those presenting with isolated dizziness/vertigo (IDV) represent a distinct phenotype characterized by a predisposition to cerebellar infarctions and a higher atherogenic lipid burden, specifically elevated LDL-C. These findings challenge the notion of a benign underlying vasculopathy in IDV stroke and underscore the necessity of comprehensive vascular assessment, including lipid profiling, in this patient population.

## 1 Background

Dizziness and vertigo are among the most common presenting symptoms in emergency departments, accounting for approximately 4% of all visits, with stroke being the underlying cause in 3–5% of these cases (1). Although an uncommon manifestation of acute stroke, patients presenting with isolated dizziness or vertigo (IDV)—defined as vestibular symptoms in the absence of other focal neurological deficits—are at a particularly high risk of misdiagnosis (2). Epidemiological studies suggest that tens of thousands of stroke patients presenting with dizziness or vertigo are misdiagnosed annually in the United States alone (1, 2), with posterior circulation infarctions often overlooked due to the non-specificity of symptoms and the limitations of standardized stroke scales like the National Institutes of Health Stroke Scale (NIHSS) in detecting subtle posterior fossa signs (3).

Although significant research efforts have been directed toward improving diagnostic accuracy (3–5), a critical knowledge gap persists. Large-scale studies confirm that vertigo is primarily associated with posterior circulation infarctions (6). However, a prospective imaging study revealed a critical paradox: vertebrobasilar stroke patients presenting with vertigo actually had significantly larger infarction volumes than those without, and a considerable proportion lacked focal deficits, highlighting that the IDV presentation may be misleadingly benign (7). This finding underscores the need to move beyond diagnosis and precisely delineate the clinical phenotype of IDV stroke compared to non-isolated dizziness/vertigo (NIDV) stroke.

A previous study by Deng et al. (8) offered initial insights, but was confined to the posterior circulation. A comprehensive comparison of infarction topography across all brain regions—coupled with a detailed analysis of atherogenic lipid profiles—between rigorously defined IDV and NIDV stroke cohorts is still lacking.

Therefore, this study aimed to systematically compare the clinical characteristics, with a specific focus on whole-brain infarction topography and lipid atherogenicity, between patients with acute cerebral infarction presenting with IDV and those with NIDV.

## 2 Methods

### 2.1 Study design and patient selection

**Figure 1:**
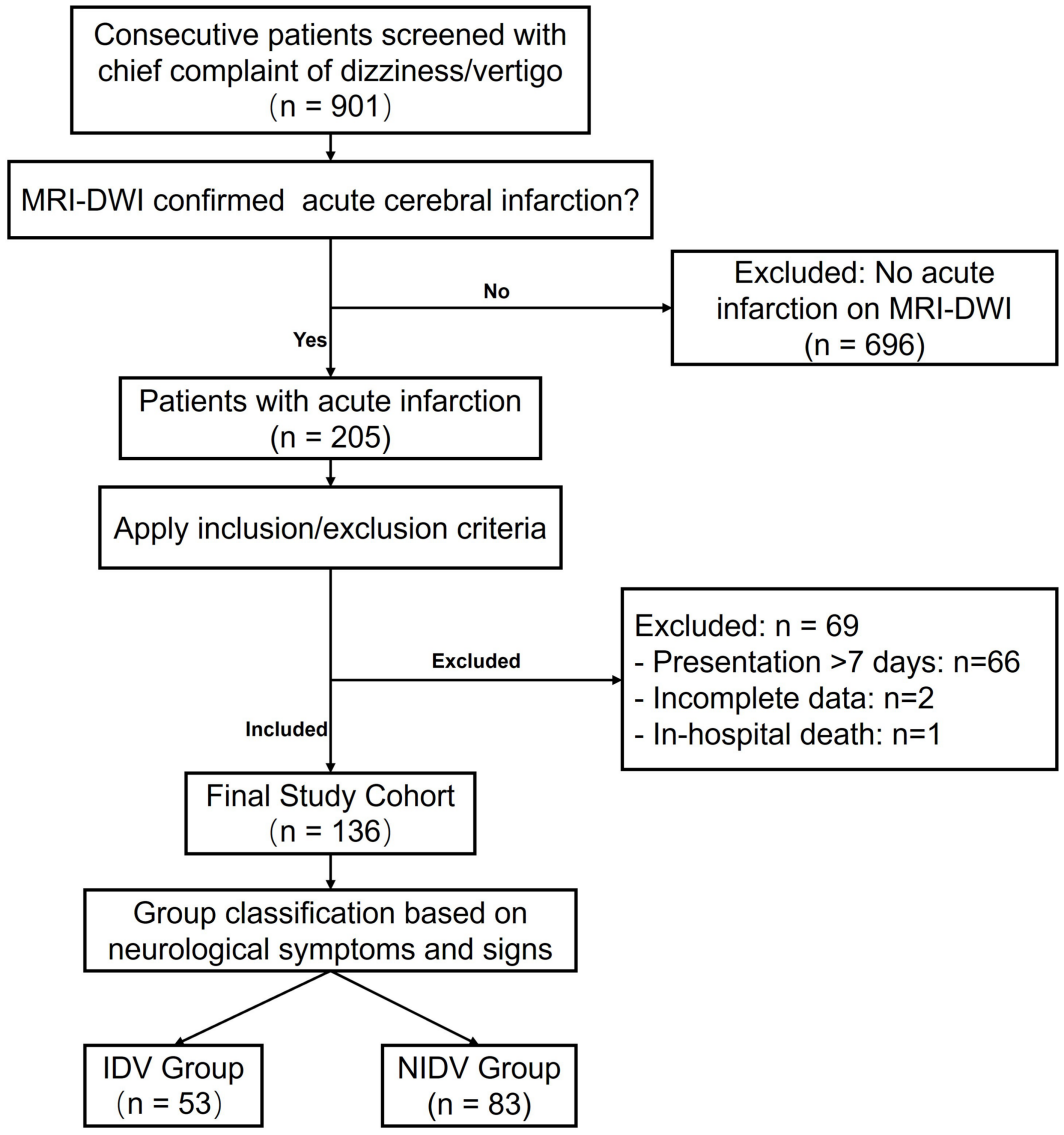
Flowchart of patient selection. A total of 901 consecutive patients presenting with dizziness or vertigo were screened. Of these, 205 patients had an acute cerebral infarction confirmed on MRI-DWI. After applying the inclusion and exclusion criteria, 69 patients were excluded (as detailed in the figure), resulting in a final study cohort of 136 patients. These patients were classified into the isolated dizziness/vertigo (IDV) group (*n* = 53) and the non-isolated dizziness/vertigo (NIDV) group (*n* = 83) based on neurological symptoms and signs.

This retrospective cohort study was conducted at the Department of Neurology, Shijiazhuang People’s Hospital. The study aimed to systematically compare the clinical characteristics, with a specific focus on whole-brain infarction topography and lipid profiles, between patients with acute cerebral infarction presenting with isolated dizziness/vertigo (IDV) and those with non-isolated dizziness/vertigo (NIDV). We utilized data from a consecutive cohort of patients presenting with dizziness or vertigo as the chief complaint between July 2022 and December 2024. The present analysis included all eligible patients with Magnetic Resonance Imaging (MRI)- Diffusion Weighted Imaging (DWI) confirmed acute cerebral infarction, irrespective of the vascular territory. The patient selection process is detailed in Figure 1.

Briefly, initial screening identified 901 patients with a chief complaint of dizziness or vertigo. Among these, 205 patients were confirmed to have an acute infarction on MRI-DWI. The final study population of 136 patients was derived after applying the following criteria: (1) Inclusion Criteria: Age ≥ 18 years, acute symptomatic cerebral infarction confirmed by MRI-DWI, and presentation to the hospital within 7 days of symptom onset; (2) Exclusion Criteria: Incomplete clinical or radiological data, presentation beyond the 7-day window, or in-hospital death from a non-stroke cause prior to data collection completion.

### 2.2 Group classification and clinical definition

Patients were classified into two groups based on a standardized review of admission symptoms and neurological signs conducted by two vascular neurologists.

The Isolated Dizziness/Vertigo (IDV) Group (*n* = 53) was defined by the presence of acute vertigo, dizziness, or gait unsteadiness as the only subjective symptom, coupled with objectively normal neurological function. This required a National Institutes of Health Stroke Scale (NIHSS) (9, 10) score of 0 points and the absence of focal neurological deficits (e.g., limb ataxia, dysmetria, motor weakness, sensory loss, or visual field defects) on a standard neurological examination.

The Non-Isolated Dizziness/Vertigo (NIDV) Group (*n* = 83) included patients presenting with dizziness or vertigo plus any other neurological symptom or objectively verified deficit (NIHSS score >0 or specific focal signs as listed above).

### 2.3 Data collection

Trained neurologists extracted data from electronic medical records using a standardized case report form. Each case was independently reviewed and confirmed by two physicians. The collected data included: (1) Demographics and Medical History: age, gender, history of hypertension, diabetes mellitus, myocardial infarction, other cardiovascular diseases, previous transient ischemic attack or ischemic stroke, smoking history, and alcohol consumption history; (2) Medication History: pre-admission use of antiplatelet agents and statins; (3) Laboratory Parameters: fasting venous blood samples were obtained within 24 h of admission. Serum lipid profiles, including total cholesterol, triglycerides, high-density lipoprotein cholesterol (HDL-C), and low-density lipoprotein cholesterol (LDL-C), were measured enzymatically using the hospital’s clinical laboratory automated biochemistry analyzer (Beckman Coulter AU5800); (4) Neuroimaging Data: The location of acute infarctions was determined based on 1.5 T head MRI and DWI findings. A predefined comprehensive classification system was used, which encompassed all supra-tentorial structures, cerebellar sub-regions (hemisphere, vermis, tonsil, flocculonodular lobe, and peduncles), and brainstem segments (midbrain, pons, medulla). For the purpose of statistical analysis and to ensure robust comparisons, anatomically or functionally proximate regions with low event rates were grouped. Specifically, the cerebellar vermis and tonsil were combined into “Other Cerebellar Regions.” Similarly, the midbrain, medulla, and cerebellar peduncles were combined into “Other Infra-tentorial Regions.

### 2.4 Statistical analysis

Continuous variables were assessed for normality using the Shapiro–Wilk test. Accordingly, normally distributed data are presented as mean ± standard deviation (SD) and were compared between groups using the independent Student’s *t*-test. In contrast, non-normally distributed data are presented as median with interquartile range (IQR) and were compared using the Mann–Whitney U test. Categorical variables were expressed as counts and percentages (%), and compared using the Chi-square test or Fisher’s exact test when expected cell frequencies were less than 5.

Binary logistic regression analysis was employed to identify factors independently associated with the stroke phenotype (IDV vs. NIDV). Variables with a *p* < 0.10 in univariate analyses were considered as candidates for the multivariate model. To mitigate potential multicollinearity, variance inflation factors (VIF) for all candidate variables were assessed prior to model building; a VIF threshold of ≥ 5 was predefined to indicate severe collinearity. In cases of severe multicollinearity among correlated variables (e.g., lipid profiles), only the variable with the greatest clinical relevance (LDL-C) was retained. The final model was constructed by entering all candidate variables simultaneously (enter method), and results are presented as adjusted odds ratios (aOR) with 95% confidence intervals (CIs).

All analyses were performed using IBM SPSS Statistics for Windows (Version 23.0; IBM Corp., Armonk, NY, United States). A two-tailed *p*-value < 0.05 was considered statistically significant.

### 2.5 Ethics statement

This study was approved by the Research Ethics Committee of Shijiazhuang People’s Hospital (Approval No.: 2025121). The committee also specifically waived the requirement for informed consent due to the retrospective nature of the study and the use of comprehensively anonymized data. All procedures were conducted in accordance with the ethical standards of the Declaration of Helsinki.

## 3 Results

### 3.1 Baseline characteristics and lesion multiplicity

A total of 136 patients were included in the final analysis, comprising 53 in the isolated dizziness/vertigo (IDV) group and 83 in the non-isolated dizziness/vertigo (NIDV) group (Figure 1). The baseline clinical characteristics and lesion multiplicity of the study participants are summarized in Table 1.

**Table 1:** Baseline clinical characteristics and lesion multiplicity of patients with acute ischemic stroke presenting with dizziness/vertigo.

Characteristic	Total (*n* = 136)	IDV group (*n* = 53)	NIDV group (*n* = 83)	*P*-value
Demographics				
Age, years, mean ± SD	65.6 ± 12.0	65.0 ± 12.0	66.0 ± 12.0	0.661
Male, n (%)	100 (73.5)	37 (69.8)	63 (75.9)	0.432
Past medical history, n (%)				
Hypertension	101 (74.3)	32 (60.4)	69 (83.1)	**0.003**
Diabetes mellitus	56 (41.2)	18 (34.0)	38 (45.8)	0.172
Myocardial infarction	9 (6.6)	4 (7.5)	5 (6.0)	0.728
Other cardiovascular diseases*	22 (16.2)	6 (11.3)	16 (19.3)	0.219
Previous TIA or ischemic stroke	48 (35.3)	18 (34.0)	30 (36.1)	0.795
Medication history, n (%)				
Antiplatelet use	29 (21.3)	8 (15.1)	21 (25.3)	0.156
Statin use	26 (19.1)	12 (22.6)	14 (16.9)	0.404
Lifestyle, n (%)				
Smoking history	53 (39.0)	18 (34.0)	35 (42.2)	0.339
Alcohol consumption history	49 (36.0)	16 (30.2)	33 (39.8)	0.257
Lipid profiles (mmol/L), mean ± SD				
Total cholesterol	4.36 ± 1.09	4.68 ± 1.71	4.16 ± 0.99	**0.006**
Triglycerides	1.47 ± 1.04	1.51 ± 1.01	1.44 ± 1.07	0.694
HDL-C	1.11 ± 0.27	1.19 ± 0.24	1.06 ± 0.28	**0.007**
LDL-C	2.85 ± 0.82	3.07 ± 0.89	2.71 ± 0.75	**0.013**
Acute lacunar infarcts, n(%)	13 (9.6)	9 (17.0)	4 (4.8)	**0.019**
Lesion multiplicity, n (%)				
Single lesion	75 (55.1)	28 (52.8)	47 (56.6)	0.664
Multiple lesions	61 (44.9)	25 (47.2)	36 (43.4)	

IDV, isolated dizziness/vertigo; NIDV, non-isolated dizziness/vertigo; SD, standard deviation; TIA, transient ischemic attack; HDL-C: high-density lipoprotein cholesterol; LDL-C: low-density lipoprotein cholesterol. *Other cardiovascular diseases exclude myocardial infarction and atrial fibrillation. *p*-values in bold indicate statistical significance (*p* < 0.05). Continuous variables were compared using independent Student’s *t*-tests. Categorical variables were compared using Chi-square tests.

The two groups were well-balanced in terms of age, gender, and most comorbidities, including diabetes, history of myocardial infarction, other cardiovascular diseases, previous transient ischemic attack (TIA) or ischemic stroke, and medication use (all *p* > 0.05). However, a history of hypertension was significantly less prevalent in the IDV group compared to the NIDV group (60.4% vs. 83.1%, *p* = 0.003). Furthermore, the IDV group exhibited a more atherogenic lipid profile at admission, with significantly higher levels of total cholesterol (4.68 ± 1.71 vs. 4.16 ± 0.99 mmol/L, *p* = 0.006), low-density lipoprotein cholesterol (3.07 ± 0.89 vs. 2.71 ± 0.75 mmol/L, *p* = 0.013), and high-density lipoprotein cholesterol (1.19 ± 0.24 vs. 1.06 ± 0.28 mmol/L, *p* = 0.007). No significant difference was observed in triglyceride levels (*p* = 0.694).

Regarding neuroimaging features, the prevalence of acute lacunar infarcts was significantly higher in the IDV group (17.0%) than in the NIDV group (4.8%) (*p* = 0.019). The prevalence of multiple acute infarctions on diffusion-weighted imaging was comparable between the IDV and NIDV groups (47.2% vs. 43.4%, *p* = 0.664), indicating a similar burden of lesion multiplicity.

### 3.2 Topographic distribution of infarctions

The analysis of infarction topography revealed distinct and clinically relevant patterns between the IDV and NIDV groups (Table 2). Notably, infarctions localized to the cerebellar hemisphere were significantly more prevalent in the IDV group than in the NIDV group (47.2% vs. 25.3%; OR = 2.636, 95% CI: 1.268–5.481; *p* = 0.009). A similar, strong association was observed for other cerebellar regions (vermis and tonsil), with lesions being more than four times as common in IDV patients (18.9% vs. 4.8%; OR = 4.593, 95% CI: 1.359–15.520; *p* = 0.009).

**Table 2:** Comparison of infarction distribution by classified brain regions.

Brain region	IDV group (*n* = 53) n (%)	NIDV group (*n* = 83) n (%)	Odds ratio (95% CI)	*P*-value
Supra-tentorial lobar regions	15 (28.3)	26 (31.3)	0.865 (0.406–1.844)	0.708
Supra-tentorial non-lobar structures	17 (32.1)	19 (22.9)	1.591 (0.736–3.440)	0.236
Cerebellar hemisphere	25 (47.2)	21 (25.3)	2.636 (1.268–5.481)	0.009
Other cerebellar regions (vermis/tonsil)	10 (18.9)	4 (4.8)	4.593 (1.359–15.520)	0.009
Pons	7 (13.2)	34 (41.0)	0.219 (0.088–0.544)	0.001
Other infra-tentorial regions (midbrain/medulla/cerebellar peduncles)	5 (9.4)	8 (9.6)	0.977 (0.302–3.161)	0.968

CI, confidence interval. *P*-values are from the Pearson Chi-Square test.

Conversely, a powerful inverse association was found with pontine infarctions. These were significantly less frequent in the IDV group compared to the NIDV group (13.2% vs. 41.0%; OR = 0.219, 95% CI: 0.088–0.544; *p* = 0.001).

No statistically significant differences were identified in the prevalence of infarctions within the supra-tentorial lobar regions, supra-tentorial non-lobar structures, or other infra-tentorial regions between the two groups (all *p* > 0.05).

### 3.3 Multivariate analysis of factors associated with IDV stroke

The multivariate binary logistic regression model identified two independent factors significantly associated with the stroke phenotype after adjusting for covariates (Table 3). Pontine infarction was a strong, significant negative predictor of the IDV phenotype (adjusted OR = 0.295, 95% CI: 0.110–0.793; *p* = 0.016). Conversely, a higher level of low-density lipoprotein cholesterol (LDL-C) was a significant positive predictor (adjusted OR = 1.669 per mmol/L increase, 95% CI: 1.035–2.690; *p* = 0.036).

**Table 3:** Multivariate binary logistic regression analysis of factors associated with isolated dizziness/vertigo (IDV) stroke phenotype.

Variable	Adjusted odds ratio	95% confidence interval	*P*-value
Pontine infarction (Yes vs. No)	0.30	0.11–0.79	0.016
LDL-C (per mmol/L increase)	1.67	1.04–2.69	0.036
Hypertension (Yes vs. No)	0.42	0.18–1.01	0.052
Cerebellar hemisphere infarction (Yes vs. No)	1.22	0.50–2.98	0.665
Other cerebellar regions infarction (Yes vs. No)	2.77	0.68–11.31	0.155

The final multivariate model was constructed using the enter method. The overall correct classification rate was 75.7%. LDL-C, low-density lipoprotein cholesterol.

A history of hypertension showed a trend toward a negative association that approached, but did not reach statistical significance (adjusted OR = 0.424, 95% CI: 0.178–1.009; *p* = 0.052). Infarctions in the cerebellar hemisphere and other cerebellar regions, which were significant in univariate analyses, were not independent predictors in the multivariate model (both *p* > 0.05). The overall model correctly classified 75.7% of cases.

## 4 Discussion

### 4.1 Principal findings

This study provides compelling evidence that acute ischemic stroke patients presenting with isolated dizziness/vertigo (IDV) represent a distinct clinical and pathophysiological subgroup compared to those with non-isolated symptoms (NIDV). The key findings are twofold. First, there is a clear divergence in infarction topography: IDV is strongly associated with cerebellar hemisphere infarctions, whereas NIDV is powerfully predicted by pontine infarction. Second, from a risk factor perspective, a higher level of low-density lipoprotein cholesterol (LDL-C) emerged as an independent predictor of the IDV phenotype, even after adjusting for infarction location and hypertension.

### 4.2 Interpretation of topographic differences

The observed topographic differences align with established neuroanatomy and stroke mechanisms. The strong association between the NIDV phenotype and pontine infarctions is anatomically rational. The densely packed pons contains critical long tracts and cranial nerve nuclei, meaning even small infarcts frequently produce the multi-modal deficits that define the NIDV presentation (11). This is consistent with studies on pontine infarction subtypes, which show that infarcts extending beyond the pons (“pontine plus” infarctions) are linked to more complex deficits and often reflect significant underlying large-artery disease (12). It is important to note that pontine infarctions can arise from heterogeneous mechanisms, including both large artery atheromatous disease (e.g., branch atheromatous disease of the basilar artery) and hypertensive small vessel disease (often corresponding to lipohyalinosis) (13). This pathophysiological heterogeneity is central to interpreting the differential risk factor profiles observed between IDV and NIDV phenotypes.

Conversely, the association of IDV with cerebellar infarctions is consistent with the cerebellum’s integral role in vestibular coordination and balance (14). The loss of independent significance for cerebellar locations in the multivariate model is best explained by the potent opposing effect of pontine infarction as a negative predictor of IDV. In effect, the presence or absence of brainstem involvement (primarily pontine) creates a fundamental clinical dichotomy. Consequently, the strong univariate association between cerebellar infarction and IDV becomes attenuated when the model accounts for the powerful confounding effect of pontine infarction.

Our findings corroborate prior evidence that infarctions in the cerebellum and/or dorsal brainstem are key predictors of vertigo in posterior circulation stroke, and a considerable proportion of such patients present without focal neurological deficits (7). This clinical scenario, where cerebellar infarction manifests with isolated symptoms, is a well-documented source of misdiagnosis due to its non-specific presentation (15). Thus, the IDV phenotype represents a genuine and high-risk clinical entity primarily linked to ischemia within eloquent cerebellar and brainstem vestibular networks.

### 4.3 LDL-C as a key predictor: from biochemical risk to topographic consequence

The most intriguing and novel finding of this study is that an elevated level of low-density lipoprotein cholesterol (LDL-C) emerged as an independent positive predictor of the isolated dizziness/vertigo (IDV) stroke phenotype, even after adjusting for infarction location and hypertension. This finding challenges any residual misconception that IDV strokes represent a “benign” or low-risk entity. Our result is strongly corroborated by a prior investigation specifically focused on patients presenting with acute vestibular syndrome (AVS), which independently identified elevated LDL-C as a significant predictor of stroke in this challenging patient population (adjusted OR = 2.54) (16). This convergence of evidence from two distinct cohorts underscores the robustness of LDL-C as a key risk factor in strokes presenting with isolated vestibular symptoms.

An additional and pathophysiologically important finding is the significantly higher prevalence of acute lacunar infarcts in the IDV group compared to the NIDV group (17.0% vs. 4.8%; *p* = 0.019). This observation, when considered alongside its independent association of IDV with elevated LDL-C, challenges a simplistic attribution to a single vascular mechanism. Classically, lacunar infarcts are linked to hypertensive cerebral small vessel disease (CSVD). However, our data suggest a more complex interplay where dyslipidemia may contribute to CSVD pathology. This concept is supported by evidence showing that genetically determined, severe elevation of LDL-C in familial hypercholesterolemia is independently associated with lacunes (17). Furthermore, early subtype-specific analyses have demonstrated that hypercholesterolemia is associated with both large vessel and small vessel stroke subtypes (18). Therefore, the co-occurrence of elevated LDL-C and a higher lacunar infarct burden in the IDV phenotype may reflect a heterogeneous vasculopathy where an atherogenic lipid profile contributes to infarction via both macro-vascular (e.g., branch atheromatous disease or artery-to-artery embolism) and micro-vascular pathways, ultimately affecting eloquent cerebellar and brainstem vestibular networks.

The pathophysiological link underlying this association is substantiated by vascular imaging studies. Research utilizing high-resolution vessel wall imaging has demonstrated that patients with posterior circulation stroke exhibit not only higher LDL-C levels but also increased basilar artery wall thickness—a direct structural marker of intracranial atherosclerosis (19). This provides a plausible mechanistic basis, suggesting that elevated LDL-C drives the atheromatous disease process within the vertebrobasilar system, the vessels supplying the brain regions critical for vestibular function.

Most compellingly, a recent large-scale spatial analysis of infarction patterns offers direct evidence of the topographic consequences of hyperlipidemia. In a cohort of over 1,200 ischemic stroke patients, high LDL-C levels were specifically associated with infarct distributions within the posterior circulation territory and resulted in significantly larger infarction volumes in this region compared to normolipidemic patients (20). This finding directly links elevated LDL-C to both the location and severity of posterior circulation infarction, providing a macroscopic clinico-radiological correlate to our biochemical finding.

Collectively, this evidence chain—spanning from clinical risk association (16) to structural vascular pathology (19), specific topographic infarct patterns (20), and now incorporating the link between severe dyslipidemia and small vessel disease (17, 18)—strongly suggests that elevated LDL-C is a key modifiable risk factor in the pathogenesis of IDV stroke, potentially acting through diverse vascular mechanisms. Therefore, the “isolated” clinical presentation likely reflects the eloquent nature of the affected cerebellar and brainstem structures within a context of significant atherosclerotic risk rather than a less severe underlying vasculopathy. Consequently, the identification of elevated LDL-C in a patient with IDV should not be dismissed but must be recognized as a marker of significant cerebrovascular risk, warranting aggressive lipid management and intensive secondary prevention strategies akin to those for stroke patients with overt neurological deficits.

### 4.4 Hypertension and the mechanistic distinction between IDV and NIDV strokes

The observed risk factor profiles offer hypothesis-generating insights into potential pathophysiological distinctions between the phenotypes. A history of hypertension was less common in the IDV group, a finding that approached statistical significance in the multivariate model. Classically, hypertension is the primary driver of cerebral small vessel disease (CSVD), which preferentially affects deep brain structures like the pons (21, 22). Notably, however, the prevalence of radiologically defined lacunar infarcts—a hallmark of CSVD—was significantly higher in the IDV group (17.0% vs. 4.8%, *p* = 0.015). This apparent discrepancy—lower hypertensive burden yet higher lacunar infarct load in IDV—challenges a purely hypertensive microangiopathic explanation for small vessel pathology in this phenotype. It is crucial to acknowledge that the use of “history of hypertension” from medical records is a limitation, susceptible to under-diagnosis and documentation bias. This finding further underscores the potential contribution of non-hypertensive mechanisms, such as lipid-mediated pathways, to small vessel pathology in the IDV cohort. Converging with this, the IDV phenotype’s independent association with elevated LDL-C points toward a predominant, lipid-driven pathophysiology. This may involve distinct aspects of intracranial large-artery atherosclerosis (e.g., branch atheromatous disease) or direct dyslipidemic injury to the cerebral microvasculature. Thus, the alignment of differential risk factor profiles (lower hypertension, higher LDL-C) with specific imaging findings (higher lacunar burden, cerebellar predilection) presents a useful framework for understanding the heterogeneity of posterior circulation stroke, positioning IDV as a phenotype where atherogenic dyslipidemia may play a central role in both macro- and micro-vascular injury.

### 4.5 Limitations

Several limitations should be acknowledged. First, the single-center retrospective design may limit generalizability of our findings. Second, the sample size, though sufficient for key findings, may have limited power for analyzing rarer infarction locations. The loss of independent significance for cerebellar infarctions in the multivariate model may be attributed to limited sample size or correlation with other included variables. Third, the lack of long-term follow-up data prevents us from determining if these phenotypic differences translate into varying recurrence risks or outcomes. Fourth, the use of “history of hypertension” as a variable, rather than standardized blood pressure measurements or ambulatory monitoring, may not capture undiagnosed or labile hypertension and is subject to documentation bias. Fifth, while magnetic resonance angiography (MRA) was available for most patients, a systematic analysis of intracranial stenosis was not performed. In this phenotype-based study, retrospectively establishing reliable causal links between specific stenoses, acute infarcts, and the clinical phenotype of dizziness across multiple vascular territories is methodologically challenging. Future prospective multicenter studies with larger samples, standardized vascular imaging, and follow-up are needed to validate these findings and explore their prognostic implications.

## 5 Conclusion

In conclusion, this study demonstrates that among patients with confirmed acute cerebral infarction, those presenting with isolated dizziness/vertigo (IDV) constitute a distinct clinical-radiological phenotype. This phenotype is characterized by a predisposition to infarctions within the cerebellar territories and is independently associated with a higher atherogenic lipid burden, specifically elevated levels of low-density lipoprotein cholesterol (LDL-C).

These findings challenge the conventional notion that an IDV presentation in the setting of confirmed stroke implies a benign or less severe underlying vasculopathy. Instead, they suggest that the “isolated” nature of the symptoms likely reflects the eloquent functional anatomy of the affected cerebellar structures—specifically responsible for vestibular function—within a spectrum of potentially heterogeneous vascular pathologies, rather than a uniformly mild vascular process.

Therefore, the identification of an IDV stroke subtype should trigger a comprehensive vascular and metabolic assessment, equivalent in rigor to that for patients with non-isolated symptoms. Given the associated atherogenic lipid profile, this assessment must include vigorous lipid profiling and management. Ultimately, these findings underscore the necessity of moving beyond a purely symptom-based risk stratification and toward a pathophysiology-driven approach for secondary prevention in all stroke patients, regardless of their initial clinical presentation.

## Data Availability

The original contributions presented in the study are included in the article/supplementary material, further inquiries can be directed to the corresponding author.
